# Electroacupuncture superiority in knee osteoarthritis: a meta-analysis of four acupuncture techniques

**DOI:** 10.3389/fmed.2025.1563715

**Published:** 2025-06-20

**Authors:** Yu Chen, Chunyan Xing, Qi Wan, Gengchun Guo, Wanlang Li

**Affiliations:** Department of Rehabilitation Medicine, Yancheng Third People’s Hospital, Yancheng, China

**Keywords:** acupuncture, electroacupuncture, knee osteoarthritis, body mass index, systematic review

## Abstract

**Background:**

Knee osteoarthritis is the most prevalent chronic joint disease affecting persons >50 years, which significantly impairs the patients’ lives. Although acupuncture can treat knee osteoarthritis; none of the studies have compared the effectiveness of four common acupuncture techniques (electroacupuncture, filiform acupuncture, warming acupuncture, and fire acupuncture) in knee osteoarthritis.

**Methods:**

The Web of Science, EMBASE, PubMed, Scopus, and CNKI databases were searched for the clinical randomized controlled trials of electroacupuncture, filiform acupuncture, warming acupuncture, and fire acupuncture in the treatment of knee osteoarthritis published before September 1, 2024. We collected 52 studies and used R software to analyze data.

**Results:**

The results of meta-analysis showed that the efficacy rates for electroacupuncture, filiform acupuncture, warming acupuncture, and fire acupuncture were 91.5, 83.4, 84.9, and 83.5%, respectively. The respective visual analog scale (VAS) scores were 2.1, 3.2, 2.9, and 4.1, respectively. Moreover, the patient’s age and body mass index (BMI) can negatively affect the efficacy rate of acupuncture therapies, whereas age and BMI positively impacts the VAS scores.

**Conclusion:**

Thus, our study suggests that electroacupuncture has the best clinical efficacy for knee osteoarthritis; however, patients’ age and BMI should be considered in future acupuncture therapies.

## Introduction

A chronic condition affecting the knee joints and their surrounding tissues, knee osteoarthritis primarily damages the articular cartilage and affects the subchondral bone as well as surrounding synovial structures (1, 2). Short-term knee osteoarthritis symptoms include pain, stiffness, reduced joint movement, and muscular weakness (3, 4). Additionally, deteriorated physical activity, worsening physical health, impaired sleep, fatigue, depression, and disability are some long-term effects (5). As the aging process quickens, more people are developing knee osteoarthritis. This is one of the major reasons for the older adult’s declining quality of life (6, 7). Therefore, more effective therapy is needed for knee osteoarthritis patients to ensure the health of aging populations.

Although drug therapy in conventional medicine may work quickly in knee osteoarthritis patients; the long-term application of these drugs can produce adverse reactions (i.e., gastric ulcer and cardiovascular disease), affecting the body’s health level (8, 9). According to traditional Chinese medicine, knee osteoarthritis is primarily caused by blood stasis and blockage. Therefore, the therapy’s major goals are eliminating blood stasis and relieving pain (10). Numerous studies have suggested that acupuncture therapy can influence the meridians system and dredge the meridians, thus promoting blood circulation, removing blood stasis, and having good curative effects on knee osteoarthritis (11–13). Moreover, electroacupuncture, filiform acupuncture, warming acupuncture, and fire acupuncture are common acupuncture therapies (14, 15). However, there are no comprehensive comparisons of the clinical efficacy of these four acupuncture therapies, which might have decreased the effectiveness of the patient’s recovery.

Furthermore, age and body mass index (BMI) can greatly influence the occurrence of knee osteoarthritis. A recent study has suggested that the prevalence of knee osteoarthritis increases with age; approximately, 15% of people >40, 50% of those >60, and up to 80% of people >75 years (16) had this condition. Because of aging-related degenerative changes in the meniscus and joint ligaments, increased bone turnover, and joint tissue calcifications, the articular cartilage becomes more susceptible to damage, thereby leading to knee osteoarthritis (17). Additionally, one study reported that a 5-unit BMI increase was associated with a 35% enhanced risk of knee osteoarthritis via a meta-analysis approach (18). Obesity increases the bearing load of knee cartilage, weakens the cartilage over time, and accelerates cartilage degeneration (19). However, how age and BMI affect acupuncture therapies for knee osteoarthritis remains ambiguous.

A recent meta-analysis synthesized evidence from 15 studies demonstrated the overall effectiveness of acupuncture in managing knee osteoarthritis (12). However, this study did not perform direct comparisons between the four specific acupuncture modalities including electroacupuncture, filiform acupuncture, warming acupuncture, and fire acupuncture. Furthermore, their analysis did not investigate potential moderators such as age and BMI that might influence therapeutic outcomes. This critical gap in existing works underscores the necessity for a comprehensive comparison of these distinct acupuncture techniques while simultaneously examining key biological factors of treatment response.

Hence, we used the meta-analytical approach to analyze 52 studies. We also asked the following questions: (1) Which acupuncture therapy has the best clinical efficacy? (2) Do osteoarthritis acupuncture therapies worsen with increasing age and BMI? Thus, we compared the efficacy rates and visual analog scale (VAS) scores of four acupuncture therapies like electroacupuncture, filiform acupuncture, warming acupuncture, and fire acupuncture. Subsequently, we explored the correlations between acupuncture therapies as well as patient’s age and VAS scores, respectively. Therefore, our study might enhance the clinical efficacy of acupuncture therapies for knee osteoarthritis and provide improved clinical guidance.

## Materials and methods

### Data collection

**Figure 1:**
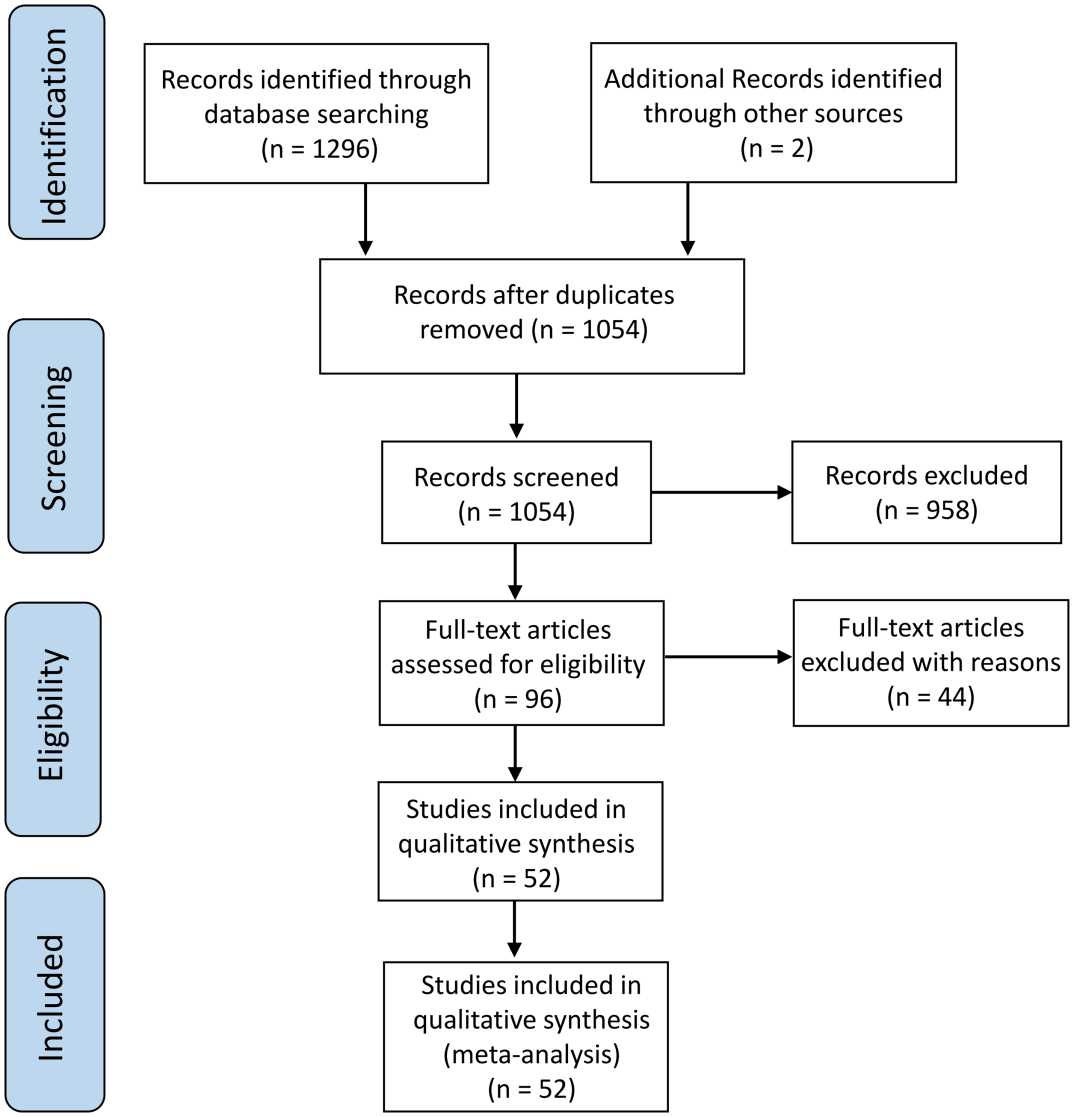
PRISMA flow chart showing the study selection procedure.

We searched the relevant published studies by using Web of Science, EMBASE, PubMed, Scopus, and CNKI databases with the following keywords: “acupuncture” OR “electroacupuncture” OR “fire acupuncture” OR “warm acupuncture” OR “filiform acupuncture” AND “knee osteoarthritis” OR “osteoarthritis.” The inclusion criteria were: (1) Studies with randomized controlled trials; (2) Participants with relevant diagnostic criteria and definite efficacy requirements; (3) Studies on at least one acupuncture therapy (electroacupuncture, filiform acupuncture, warming acupuncture, or fire acupuncture); and (4) Those with primary outcome measures of efficacy rates and VAS scores. Notably, after receiving acupuncture therapy, patients’ pain and VAS scores decreased, suggesting good patient recovery. Finally, we selected 52 studies as per the PRISMA guidelines (Figure 1). We also collected data about patients’ age and BMI to explore their influences on efficacy rates and VAS scores, respectively. Besides, we extracted the number of patients, gender, Kellgren-Lawrence grade (II and III grades), treatment duration (min), and intervention details to demonstrate baseline characteristics of included studies (Table 1).

**Table 1:** Baseline characteristics of included studies.

Characteristics	Electroacupuncture	Filiform acpuncture	Warming acupuncture	Fire acupuncture	Significance on effective rates (*p*)	Significance on VAS score (*p*)
Patients number	84.6 ± 31.5	82.9 ± 43.8	79.8 ± 21.7	81.2 ± 15.4	0.781	0.694
Female (%)	38.1 ± 4.5	40.5 ± 5.2	36.6 ± 5.1	41.2 ± 6.3	0.439	0.328
K-L grade III (%)	71.3 ± 8.5	70.9 ± 9.8	68.8 ± 6.9	69.7 ± 8.4	0.214	0.367
Treatment duration	25.0 ± 8.2	23.5 ± 7.2	26.8 ± 6.4	21.8 ± 9.4	0.473	0.536

Value are expressed as means ± standard deviation. There exist no significant differences for baseline characteristics among four acupuncture techniques, and these characteristics had no significant influences on effective rates and VAS scores.

Moreover, all included patients had primary knee osteoarthritis, and no participants had received prior osteoarthritis-specific treatments (including medications, injections, or surgery). Acupuncture techniques were administered without patient-specific selection criteria, enabling direct comparison of their therapeutic effects in this homogeneous population.

Our data encompassed four distinct acupuncture techniques, each with unique therapeutic characteristics: (1) Electroacupuncture combined traditional needle insertion with low-frequency electrical stimulation to enhance neuromodulatory effects; (2) Filiform acupuncture utilized standard manual manipulation of thin sterile needles to elicit *Deqi* sensations without adjunctive stimulation; (3) Warming acupuncture integrated moxibustion-derived thermal stimulation by burning purified mugwort on needle handles, creating synergistic thermal and mechanical effects; and (4) Fire acupuncture involved brief punctures with pre-heated needles to induce localized microtrauma and thermal regulation.

### Statistical analysis

We used ANOVA analysis to compare the clinical efficacies, i.e., the efficacy rates and VAS scores of electroacupuncture, filiform acupuncture, warming acupuncture, and fire acupuncture techniques. Furthermore, linear regression models helped to explore the associations between patients’ age and BMI as well as primary outcome measures. We used the Shapiro–Wilk test to assess the normality of the models’ residuals (20). Our results confirmed that all models exhibited normal distributions. Finally, there is no publication bias in our models via Egger’s test using *metafor* package (Supplementary Table S1). All statistical analyses were performed using the R platform (21) with the *ggplot2* package (22).

## Results

Out of 52 studies, 11, 19, 13, and 9 dealt with electroacupuncture, filiform acupuncture, warming acupuncture, and fire acupuncture, respectively. Furthermore, 95% of the studies on acupuncture therapy’s impact on knee osteoarthritis have been conducted from 2020 to 2024. These trials studies also included a wide range of patients’ efficacy rates (71.4–96.7%, mean ± SD = 85.5% ± 6.1%), VAS scores (1.4–4.6, mean ± SD= 3.1 ± 0.9), age (35–77, mean ± SD= 57.0 ± 10.4), and BMI (17.9–31.9, mean ± SD= 22.2 ± 3.5), respectively. Patients’ ages ranged from 35 to 64, 37 to 70, 44 to 75, and 50 to 77 for electroacupuncture, filiform acupuncture, warming acupuncture, and fire acupuncture techniques, respectively. Similarly, the respective BMIs ranged from 17.9 to 25.3, 18.2 to 28.9, 18.3 to 31.9, and 20.1 to 25.4, respectively.

**Figure 2:**
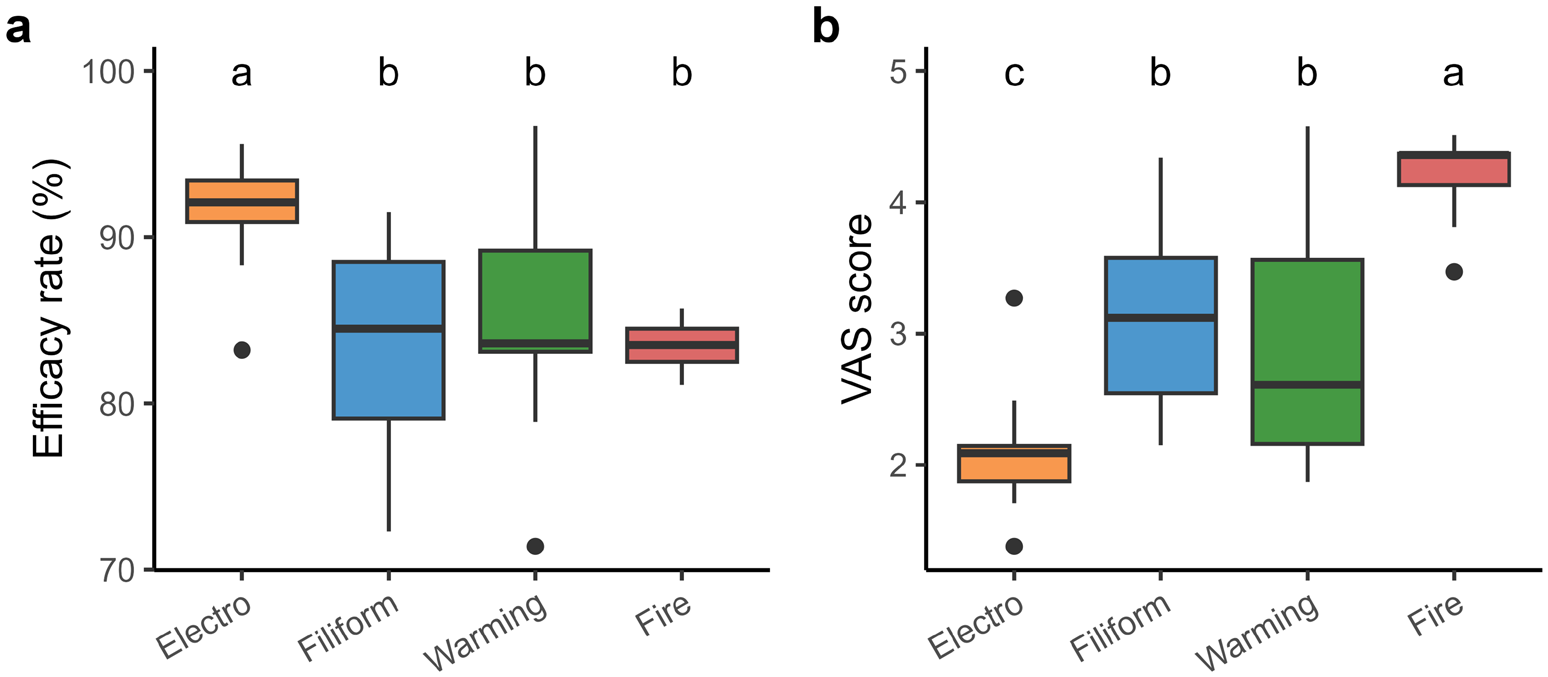
Comparisons between the clinical efficacies of different acupuncture therapies. **(a)** Effective rates, and **(b)** Visual analog scale (VAS) scores. Different lowercase letters indicate significance among acupuncture therapies.

Baseline characteristics were balanced across acupuncture techniques, with no significant differences in number of patients, sex distribution, Kellgren-Lawrence grade, or treatment duration, and these variables had no significant effects on efficacy rates and VAS scores (all *p* > 0.05; Table 1). This homogeneity supports the validity of direct efficacy comparisons among acupuncture techniques. Across all studies, mean efficacy rate values for electroacupuncture, filiform acupuncture, warming acupuncture, and fire acupuncture were 91.5, 83.4, 84.9, and 83.5%, respectively (Figure 2a). Although the efficacy rate of electroacupuncture was higher than that of filiform acupuncture, warming acupuncture, and fire acupuncture, the efficacy rates did not differ significantly among filiform acupuncture, warming acupuncture, and fire acupuncture methods. Additionally, the mean VAS scores for electroacupuncture, filiform acupuncture, warming acupuncture, and fire acupuncture were 2.1, 3.2, 2.9, and 4.2, respectively. However, electroacupuncture had a lower VAS score than filiform acupuncture, warming acupuncture, and fire acupuncture (Figure 2b).

**Figure 3:**
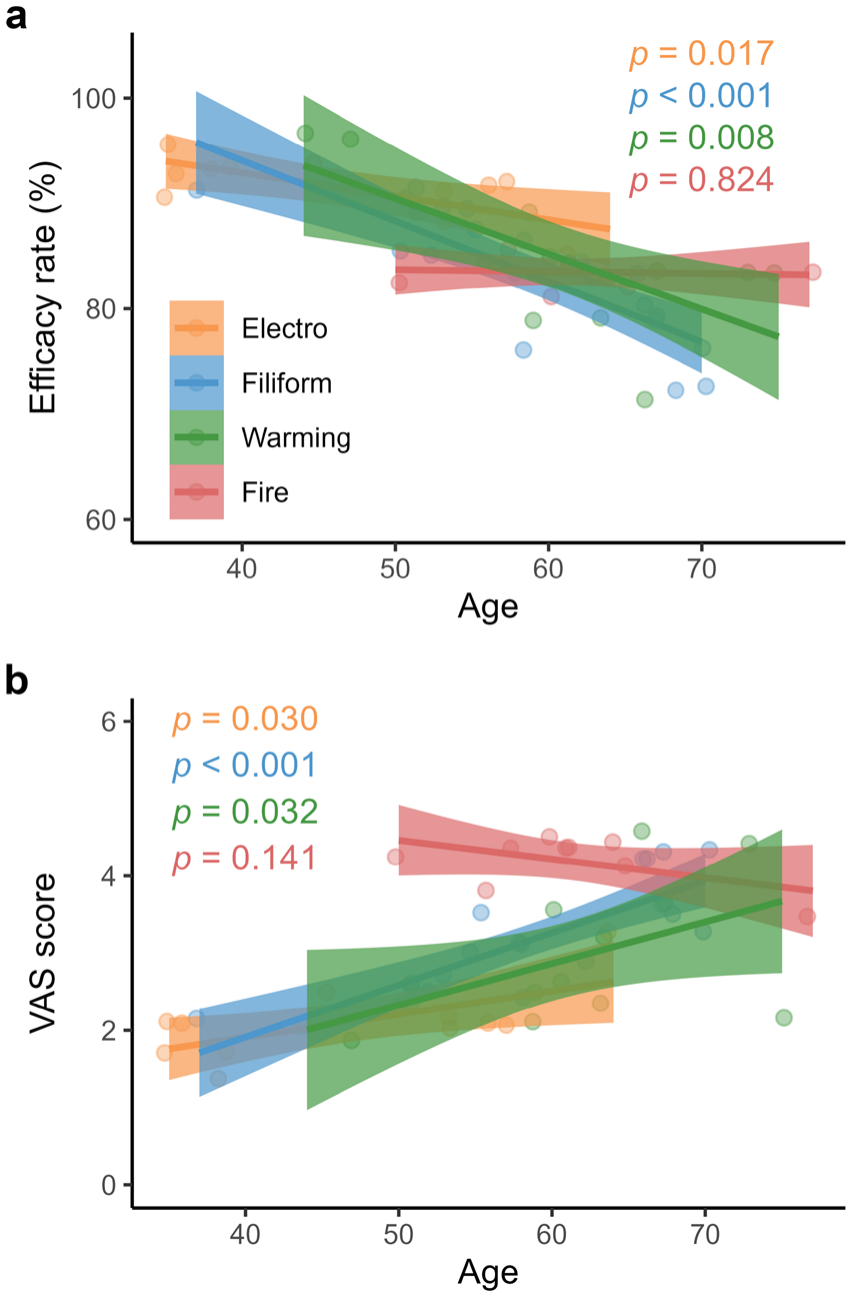
Associations between the clinical efficacies of various acupuncture therapies and age. **(a)** Effective rates (electroacupuncture: y = −0.22x + 101.80; filiform acupuncture: y = −0.57x + 117.03; warming acupuncture: y = −0.52x + 116.70, and fire acupuncture: y = −0.02x + 84.60), and **(b)** Visual analog scale (VAS) scores (electroacupuncture: y = 0.03x + 0.71; filiform acupuncture: y = 0.07x – 0.79; warming acupuncture: y = 0.05x – 0.36, and fire acupuncture: y = −0.02x + 5.68). Colored lines and shaded areas represent the fitted lines and 95% confidence intervals, respectively. Supplementary Table S2 demonstrates detailed information about regression models.

**Figure 4:**
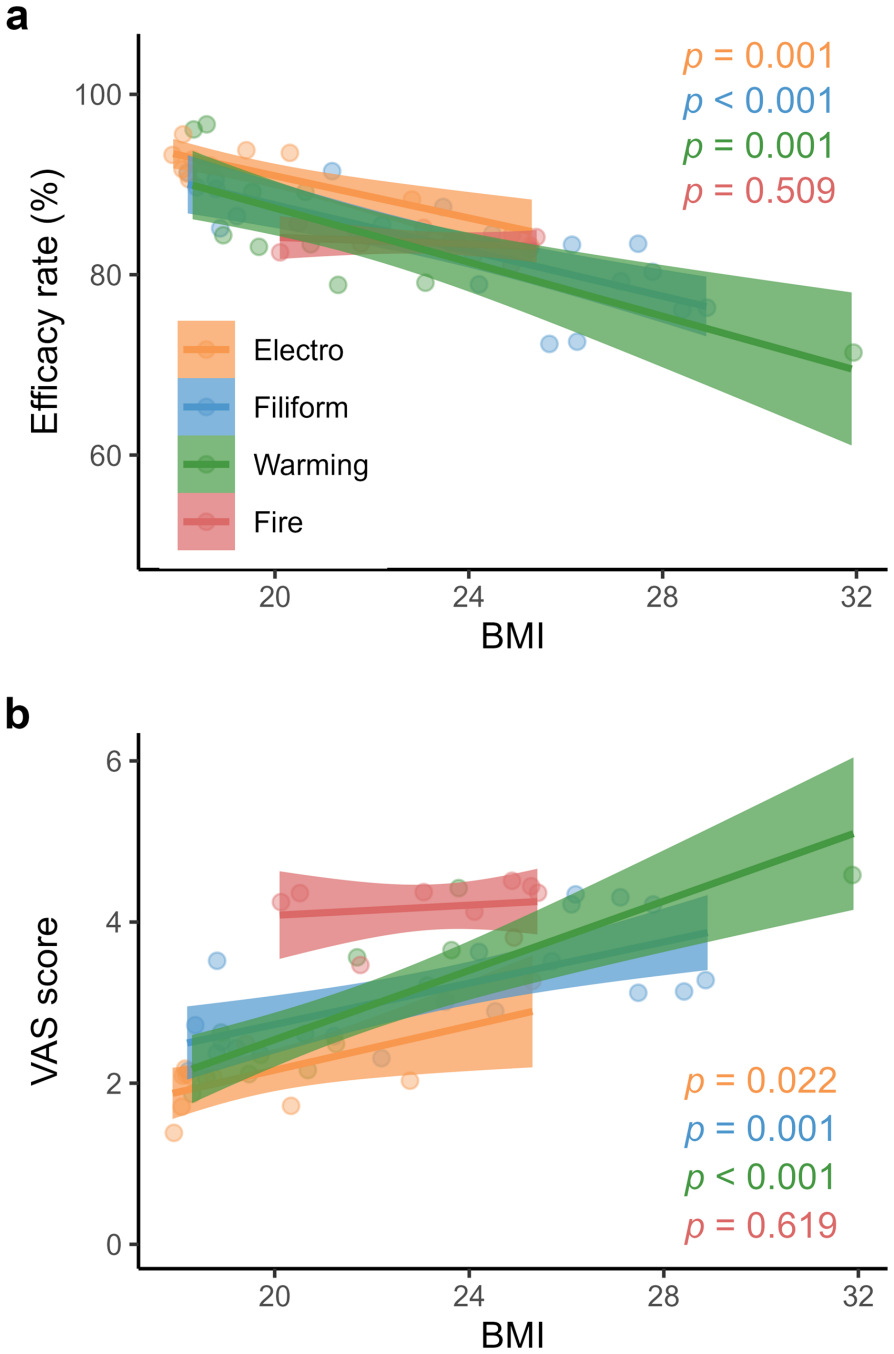
Relationships between the clinical efficacies of various acupuncture therapies and BMI. **(a)** Effective rates (electroacupuncture: y = −1.17x + 114.33; filiform acupuncture: y = −1.27x + 113.07; warming acupuncture: y = −1.50x + 117.37, and fire acupuncture: y = −0.19x + 87.85), and **(b)** Visual analog scale (VAS) scores (electroacupuncture: y = 0.14x – 0.59; filiform acupuncture: y = 0.13x + 0.17; warming acupuncture: y = 0.21x – 1.76, and fire acupuncture: y = 0.03x + 3.45). Colored lines and shaded areas represent the fitted lines and 95% confidence intervals, respectively. Supplementary Table S3 provides comprehensive details regarding regression models.

While the efficacy rates of electroacupuncture, filiform acupuncture, and warming acupuncture decreased significantly with patients’ age (Figure 3a; Supplementary Table S2), corresponding VAS scores increased with their age (Figure 3b; Supplementary Table S2). Similarly, patients’ BMI negatively impacted the efficacy rates of electroacupuncture, filiform acupuncture, and warming acupuncture (Figure 4a; Supplementary Table S3) while having positive impacts on their VAS scores (Figure 4b; Supplementary Table S3). Moreover, the efficacy rate and VAS score of fire acupuncture did not change significantly with patient’s age and BMI.

## Discussion

### Clinical efficacy of four acupuncture therapies

Four acupuncture therapies (electroacupuncture, filiform acupuncture, warming acupuncture, and fire acupuncture) were significantly beneficial in knee osteoarthritis cases (Figures 2a,b). Consistent with the recent meta-analyses (23, 24), our results demonstrated that acupuncture significantly improves the symptoms of knee osteoarthritis more effectively than other therapies. This could be an outcome of acupuncture’s ability to reduce peripheral nerves’ excitability, relieve muscle spasms, improve blood circulation, and alleviate knee pain (12). Furthermore, the working mechanisms of techniques like electroacupuncture, filiform acupuncture, warming acupuncture, and fire acupuncture involve activating and reducing CB2 receptor and interleukin-1β expressions (25), improving blood circulation and nourishing joints (26) as well as removing disperse knots and enhancing joint movement by applying heat to the knee joint (27), respectively. They also delay cartilage deterioration by regulating the extracellular matrix degradation and affecting the Wnt signaling pathway (28), respectively. Thus, our findings, together with other outcomes (29) suggest that acupuncture is highly effective for knee osteoarthritis.

Based on previous meta-analyses (12, 23), our study compared the efficacy rates and VAS scores of electroacupuncture, filiform acupuncture, warming acupuncture, as well as fire acupuncture, and demonstrated that electroacupuncture is the most effective therapy for knee osteoarthritis (Figure 2a). Electroacupuncture is combined with electrical stimulation, filiform acupuncture is mechanical stimulation, warming acupuncture is thermal effect, and fire acupuncture is thermal trauma. These different physical stimuli lead to different physiological responses, such as nerve activation, inflammation inhibition, and improved blood circulation, which in turn produce different therapeutic effects (14). Moreover, electroacupuncture achieves both neuromodulatory and anti-inflammatory effects through precisely controlled electrical impulse parameters, and the depth and range of stimulation are more controllable, leading to more stable therapeutic outcomes (30, 31).

### Moderators of acupuncture therapies for knee osteoarthritis

While efficacy rates of acupuncture therapies decreased with age, respective VAS scores increased as patients aged. Based on the fitted lines, the efficacy rates of electroacupuncture, filiform acupuncture, and warming acupuncture decreased from 90.8 to 86.4%, 88.5 to 77.1%, and 90.7 to 80.3%, respectively, from the 5th percentile (50 years) to the 95th percentile (70 years). In contrast, their corresponding VAS scores increased from 2.2 to 2.8, 2.7 to 4.1, and 2.1 to 3.1, respectively (Figures 3a,b). These results suggest that the clinical efficacies of acupuncture therapies are mostly age-dependent and decline with patient age. This might be due to the fact that knee tissue’s ability to adapt to biomechanical damage weakens with age, leading to cellular senescence and sarcopenia (17).

Our results also demonstrated that the clinical efficacies of acupuncture therapies decreased with patients’ BMI (Figure 4a). The effective rates of electroacupuncture, filiform acupuncture, and warming acupuncture decreased by 14.2, 15.9, and 19.1%, respectively, from the 5th percentile (18.9) to the 95th percentile (30.1) of BMI. In contrast, corresponding VAS scores increased by 43.1, 43.6, and 51.5%, respectively. The probable explanation is that obesity increases the knee joint’s biomechanical load, aggravates cartilage injury, and results in articular cartilage degeneration as well as bone spur formation (32). However, we did not find any significant relationship between fire acupuncture, and age as well as BMI; this may be attributed to the smaller sample size of fire acupuncture, i.e., only nine studies. Therefore, we advocate additional research on fire acupuncture technique in such patients.

### Suggestions for clinical therapy

Knee osteoarthritis is the most common chronic joint disease for individuals >50 years, which significantly impairs patients’ quality of life (7). Additionally, knee osteoarthritis patients experience significant financial strain, which can significantly impact the social economy, and attract global attention (33). Our study suggests that the clinical efficacies of acupuncture therapies are significantly associated with the patient’s age and BMI. These factors should be considered when providing clinical therapy. For example, electroacupuncture should be considered preferentially in a 60-year-old patient with a BMI > 25. Furthermore, 19.1% of the patients were <50 years old, indicating that younger people typically suffer from knee osteoarthritis. Therefore, adults are advised to maintain a healthy weight through physical exercise and low-calorie diet to prevent knee osteoarthritis.

## Conclusion

In summary, this study indicates that acupuncture has a good curative effect on knee osteoarthritis. Electroacupuncture is the most effective curative therapy for knee osteoarthritis and may be used as an optimization strategy for its clinical management. Moreover, age and BMI are significant moderators of the effects of acupuncture therapies for knee osteoarthritis, which deserves clinical attention.

## Data Availability

The datasets presented in this study can be found in online repositories. The names of the repository/repositories and accession number(s) can be found in the article/Supplementary material.
