# Supplementary material for: Electroacupuncture superiority in knee osteoarthritis: a meta-analysis of four acupuncture techniques
Source: Front Med (Lausanne). 2025 Jun 20;12:1563715. doi: 10.3389/fmed.2025.1563715 (PMC12226530; doi:10.3389/fmed.2025.1563715)
Supplement: Supplementary file 1 [file Data_Sheet_1.docx]

**Supplementary Table 1.** Results from Egger's tests of publication bias on our models. The *p*-value above 0.05 suggests there is no publication bias.

| Acupuncture | *z* | *p* |
| --- | --- | --- |
| Electroacupuncture | 0.16 | 0.892 |
| Filiform acupuncture | 0.04 | 0.931 |
| Warming acupuncture | 0.13 | 0.882 |
| Fire acupuncture | 0.09 | 0.917 |

**Supplementary Table 2**. The effect (degrees of freedom, F and *p* values) of age on efficacy rate and VAS score. Significance is in bold.

| Acupuncture | Slope | df | F | *p* |
| --- | --- | --- | --- | --- |
| **Efficacy rate** | | | | |
| Electroacupuncture | -0.22 | 1,9 | 8.55 | **0.017** |
| Filiform acupuncture | -0.57 | 1,17 | 33.38 | **<0.001** |
| Warming acupuncture | -0.52 | 1,11 | 10.56 | **0.008** |
| Fire acupuncture | -0.02 | 1,7 | 0.05 | 0.824 |
| **VAS score** | | | | |
| Electroacupuncture | 0.03 | 1,9 | 0.05 | **0.030** |
| Filiform acupuncture | 0.07 | 1,17 | 33.79 | **<0.001** |
| Warming acupuncture | 0.05 | 1,11 | 4.60 | **0.032** |
| Fire acupuncture | -0.02 | 1,7 | 2.76 | 0.141 |

**Supplementary Table 3**. The effect (degrees of freedom, F and *p* values) of BMI on efficacy rate and VAS score. Significance is in bold.

| Acupuncture | Slope | df | F | *p* |
| --- | --- | --- | --- | --- |
| **Efficacy rate** | | | | |
| Electroacupuncture | -1.17 | 1,9 | 20.92 | **0.001** |
| Filiform acupuncture | -1.27 | 1,17 | 28.51 | **<0.001** |
| Warming acupuncture | -1.50 | 1,11 | 17.62 | **0.001** |
| Fire acupuncture | -0.19 | 1,7 | 0.49 | 0.509 |
| **VAS score** | | | | |
| Electroacupuncture | 0.14 | 1,9 | 7.63 | **0.022** |
| Filiform acupuncture | 0.13 | 1,17 | 14.73 | **0.001** |
| Warming acupuncture | 0.21 | 1,11 | 29.14 | **<0.001** |
| Fire acupuncture | 0.03 | 1,7 | 0.27 | 0.619 |
